# Intraspinal Cocainisation

**Published:** 1904-05-14

**Authors:** 


### INTRASPINAL COCAINISATION.

Dr. Alfred S. Gubb[1] considers that racchis cocainisation has many advantages over general anæsthesia when operations are to be performed on the lower extremities. The advantages which this method possesses are its simplicity, the readiness with which a patient is prepared for the intended operation, the absence of struggling, of respiratory disturbance, and of subsequent discomfort. The patient sits on the operating table and after the skin over the lumbar region of the spine has been disinfected, a sterilised needle connected with a syringe is plunged two inches into the back in the space above the last lumbar vertebra. The syringe is disconnected, cerebro-spinal fluid drips for a minute, then $\frac{1}{3}$ grain of cocaine in solution is injected; after five minutes the lower limbs are insensitive to cutting, though not to pressure, and remain so for 30 minutes or more. Occasionally there is a little vomiting. To illustrate the slight constitutional effects of the injection and the total absence of shock, Dr. Gubb mentions a case of operation for hernia where the patient, who had been anæsthetised in this way left the theatre smiling and asked if she might be allowed a square meal as she was hungry.

[1] Brit. Med. Jour., April 23.

